# Calendar

**DOI:** 10.1155/S1463924678000176

**Published:** 1978


figured as a communications concentrator for a Honeywell 6000 host computer. The system presently provides 55 terminals with a two-to-five-second response time. The medical centre can expand the system with the simple addition of terminals, cabling and minor additions to the NOVA, without increasing the terminal response time.

Communications between the NOVA 3 and the Honeywell 6000 host is accomplished synchronously on two 9.6 K-baud lines. The NOVA 3 can communicate asynchronously with up to 75 DASHER display terminals and an additional 80 printers.

Approximately half of the terminals are located in doctors' clinics in the medical centre, where they are used for professional billing, retrieving pertinent patient information, and verifying insurance claim information. Since each clinic operates as an individual entity as part of the Bowman Gray School of Medicine, the computer system must handle 26 different clinics staffed by a total of 130 doctors, each of whom can bill patients.

Future plans at the medical centre are to add on-line admissions, medical record retrieval, dietary restrictions, laboratory tests, and other departments to the system, and to use some of the resulting data for statistical surveys.

### Automatic methods of analysis

A summer school on Automatic Methods of Analysis is being organised by the Chemical Society, UK at Swansea, Wales, July 9-13 1979. Automation is receiving increasing interest in analytical chemistry. This summer school is designed to acquaint laboratory managers, technical staff etc. with both the recent developments in the field as well as with those important aspects of background philosophy and the economic rationale. In addition to lectures, the residential course will provide laboratory sessions in which the student can use a range of the most up to date equipment. There will also be tutorial sessions and a group problem will be set, and students will carry out a systems study on a real and complicated analytical problem. Experience of the laboratory, who have calculated the problem will be made available to participants so that they can discuss in depth the various strategies. Lectures and tutorials will be given by international experts in automation.

Further information is available from D. G. Porter, Laboratory of the Government Chemist, Cornwall House, Stamford Street, London SE1 9NG, UK.

### Calendar

### 1978

**Automation in Microbiology**
November 20-21, Chicago, USA.
*Robert S. First Inc., 405 Lexington Avenue, New York, NY 10017, USA.*

**Automation and Atomic Spectroscopy**
December 7, London, UK.
*D. Porter, Laboratory of the Government Chemist, Cornwall House, Stamford Street, London SE1 9NQ, UK.*

**8th Technicon International Congress: Laboratory Management and Automation**
December 12-14, London, UK.
*Technicon Instruments Co. Ltd., Evans House, Hamilton Close, Houndmills, Basingstoke, RG21 1BZ, Hampshire, UK.*

### 1979

**6th Annual Reports on Analytical Atomic Spectroscopy Symposium**
January 4, Sheffield, UK.
*Miss P. E. Hutchinson, The Chemical Society, Burlington House, London W1V 0BN, UK.*

**Optical Data Display Processing & Storage**
January 23-26, Orlando, Florida, USA.
*Society of Photographic Scientists and Engineers, 1411 K Street, NW, Suite 930, Washington, D.C. 20005, USA.*

**Microsystems '79: an overview of microprocessor technology and applications**
January 31-February 2, London, UK.
Iliffe Promotions Ltd., Dorset House, Stamford Street, London, SE1 9LU, UK.

**The Applications of Microprocessors: A review lecture by Professor J. H. Westcott**
February 8, London, UK.
*The Executive Secretary, The Royal Society, 6 Carlton House Terrace, London SW1Y 5AG, UK.*

**Use of the Microprocessor as a Component in Measurement and Control Equipment**
February 13-15, Stevenage, Herts., UK.
*Carole Meads, Sira Institute, South Hill, Chislehurst, Kent, UK.*

**30th Annual Conference on Analytical Chemistry and Applied Spectroscopy**
5-9 March, Cleveland, USA.
*Mrs. L. Briggs, 437 Donald Road, Pittsburgh, Passadena 15235, USA.*

**International Conferences on Spectroscopy**
29-30 March, Miami, USA.
*Vijay Mohan Bhatnagar, Alena Enterprises of Canada, P.O. Box 1779, Cornwall, Ontario, Canada K6H 5V7.*

**Kongress der Deutschen Gesellschaft fur Laboratoriumsmedizin**
March 29-April 3, Berlin, Germany.
*Wilhelm Syborg, Kongresshall Berlin, John-Foster-Dulles-Allee 10, 1000 Berlin 21, West Germany.*

**3rd International Symposium on Capillary Chromatography**
April 30-May 3, Hindelang/Allgau, W. Germany
*R. E. Kaiser, P.O. Box 1308, D-6702 Bad Durkheim 1, Germany.*

**3rd European Congress of Clinical Chemistry**
June 4-8, Brighton, UK.
*Dr P. J. N. Howarth, Department of Chemical Pathology, Guy's Hospital, Medical School, London, SE1 9RT, UK.*

**2nd World Chromatography Conference**
June 5-6, Lisbon, Portugal.
*V. M. Bhatnagar, P.O. Box 1779, Cornwall, Ontario K6H 5V7, Canada.*

**10th International Symposium on Chromatography and Electrophoresis**
June 19-20, Venice, Italy.
*Dr Alberto Frigerio, Instituto di Riccerche Farmacologiche "Mario Negri", Via Eritrea 62, 20157 Milan.*

**8th International Conference on Atomic Spectroscopy**
July 1-6, Cambridge
*Association of British Spectroscopists, P.O. Box 109, Cambridge, CB1 2HY, UK.*

**2nd Canadian World Chromatography Conference**
July 5-6, Toronto, Canada.
*V. M. Bhatnagar, P.O. Box 1779, Cornwall, Ontario K6H 5V7, Canada.*

**Summer School on Automatic Methods of Analysis**
July 9-13, Swansea, UK.
*D. Porter, Laboratory of the Government Chemist, Cornwall House, Stamford Street, London SE1 9NQ, UK.*

**Analysis '79: Automation in Industrial and Clinical Chemistry.**
July 16-18, London, UK.
*Scientific Symposia Ltd., 33-35 Bowling Green Lane, London, EC1R 0DA, UK.*

